# Dominance of *Enterobacter* spp. in Asymptomatic Sweet Potato Tubers Grown in Foot Rot–infested Fields

**DOI:** 10.1264/jsme2.ME26007

**Published:** 2026-06-03

**Authors:** Chiyomi Kubota, Megumi Hashimoto, Yasutoshi Ougitani, Rinka Yujima, Matsujiro Ishibashi, Hirohito Tsurumaru

**Affiliations:** 1 Applied Microbiology Laboratory, Faculty of Agriculture, Kagoshima University, 1–21–24 Korimoto, Kagoshima, 890–0065, Japan; 2 Graduate School of Agriculture, Forestry and Fisheries, Kagoshima University, Kagoshima, Japan; 3 Department of Agriculture, Kagoshima Prefectural Government, Kagoshima, Japan; 4 The United Graduate School of Agricultural Sciences, Kagoshima University, Kagoshima, Japan

**Keywords:** Bacterial community analysis, sweet potato, *Enterobacter*

## Abstract

Since 2018, foot rot disease caused by *Diaporthe destruens* has emerged as a serious threat to sweet potato production in Japan. Although plant-associated bacteria have been suggested to contribute to disease suppression, the bacterial community structure of asymptomatic sweet potato tubers in foot rot–infested fields remains unclear. In the present study, we analyzed the bacterial communities of asymptomatic tubers collected adjacent to infected tubers from four foot rot–infested fields. Thirteen bacterial taxa with >1% relative abundance were detected, among which *Enterobacter* (ASV-129 sequence; 427 bp) was dominant, accounting for an average of 49.4% of total 16S rRNA gene amplicon reads. Forty bacterial strains were isolated from asymptomatic sweet potato tubers using R2A agar plates. Among them, strain 12CK showed 100% sequence identity to ASV-129 and was identified as *Enterobacter hormaechei* based on a genome anal­ysis (genome size: 4,832,483 bp). In the present study, *D. destruens* 2YO was also isolated from infected plants, and its pathogenicity was confirmed. In growth inhibition assays (dual culture assay), *E. hormaechei* 12CK strongly inhibited the growth of *D. destruens* 2YO. Pot and field trials will be required to examine the applicability of strain 12CK as a microbial biocontrol agent.

Sweet potato (*Ipomoea batatas* [L.] Lam.) is an important crop in Kagoshima Prefecture, Japan. In 2023, its production reached 215,000 tons, the highest in the country, accounting for approximately 30% of Japan’s total output. In Kagoshima Prefecture, sweet potato tubers are widely used as the base ingredient of the distilled spirit shochu (42%), for starch production (39%), processed foods (9%), and meals (9%) (https://www.alic.go.jp/content/001232563.pdf [in Japanese; accessed September 2025]). In recent years, sweet potato production has been negatively affected by the widespread occurrence of foot rot disease caused by *Diaporthe destruens*. This disease was first described in the United States in 1912 and has subsequently been reported in Taiwan in 2012, China in 2014, Korea in 2015 (Gai *et al.*, 2016), and in the southern region of Japan (Okinawa, Kagoshima, and Miyazaki Prefectures) in 2018 (Fujiwara *et al.*, 2021; Maeda *et al.*, 2022), where it is currently prevalent.

Current management practices for foot rot disease include the removal of infected plant residues, soil disinfection, and the use of disease-free planting materials. The development of resistant cultivars and new agrochemicals has also been pursued; however, these approaches are often constrained by long breeding periods and environmental concerns associated with chemical inputs. In this context, plant-associated microorganisms have attracted increasing attention because of their potential roles in disease suppression and ecosystem sustainability. Previous studies demonstrated that bacteria isolated from sweet potato tissues may inhibit *D. destruens in vitro*, and their application has reduced disease severity in pot experiments. Genomic anal­yses further suggested that these bacteria harbor genes related to the production of antimicrobial metabolites (Mateus *et al.*, 2021). From a microbial ecological perspective, successful disease suppression by beneficial microbes depends not only on antagonistic activity, but also on the ability of introduced or indigenous microbes to colonize and persist in plant-associated niches (Wu *et al.*, 2019). Although fluorescent tagging techniques provide detailed insights into bacterial colonization, they are labor-intensive and not always suitable for large-scale screening. Therefore, the relative abundance of microbes within plant tissues or the rhizosphere has been proposed as a practical indicator for colonization ability. Microbial populations associated with asymptomatic plants located near diseased individuals may reflect communities adapted to competitive and pathogen-rich environments, making them promising sources of candidate beneficial microbes (Cao *et al.*, 2020).

Previous studies reported that *Pseudomonas*, *Enterobacteriaceae*, *Erwinia*, and *Burkholderia* are dominant bacterial taxa associated with sweet potatoes grown in soil environments (Puri *et al.*, 2019). In the present study, we investigated the bacterial community associated with asymptomatic sweet potatoes growing adjacent to plants infected with foot rot disease in the field. Dominant bacterial strains were isolated, and their antagonistic activity against *D. destruens* was evaluated. In addition, a simple assay for assessing the pathogenicity of *D. destruens* was established. This study provides ecological insights into sweet potato–associated bacterial communities and identifies bacterial candidates with potential relevance to the biological control of foot rot disease.

## Materials and Methods

### Sweet potato

Sweet potato tubers were harvested on 10 November 2018 from four *D. destruens*–infected fields (designated AH, BH, CH, and DH) managed by two farmers in Minamisatsuma city, Kagoshima Prefecture, Japan. The cultivation of sweet potato in these fields followed standard local production practices in Kagoshima Prefecture. Chemical fertilizers were applied at rates of ≤5, 16.5, and 16.5 kg 10a^–1^ for nitrogen (N), phosphorus (P), and potassium (K), respectively. Ridges were formed, covered with plastic mulch, and subjected to soil disinfection. The ridge width was 80–90 cm, and the planting distance between seedlings was 30–45 cm. Sweet potato seedlings were transplanted by May. Asymptomatic sweet potato tubers were collected from six plants adjacent to diseased plants in each field, and all tubers attached to the underground stems of each plant were used. All tubers were thoroughly washed with tap water to remove adhering soil. Diseased sweet potatoes were used for the isolation of *D. destruens*. Asymptomatic sweet potatoes were processed separately and homogenized using a blender (OSTER model 4090-J; Jarden Consumer Solutions). The resulting powders were stored at –80°C for later use.

### Microbial community anal­ysis of asymptomatic sweet potato

Bacterial cells, including both epiphytic and endophytic populations, were extracted from 50 g of pooled asymptomatic sweet potato powder without cultivation, according to the method of Ikeda *et al.* (2009). Metagenomic DNA was subsequently extracted from the recovered bacterial cell fraction. DNA samples were submitted to Bioengineering Lab. for amplicon sequencing using the Illumina MiSeq platform. The V3-V4 region of the bacterial 16S rRNA gene was amplified using the primers 341F (5′-CCTACGGGNGGCWGCAG-3′) and 805R (5′-GACTACHVGGGTATCTAATCC-3′) (Herlemann *et al.*, 2011). Paired-end sequencing was performed with a read length of 2×300 bp. Sequence data were processed and analyzed using QIIME 2 version 2022.11 (Bolyen *et al.*, 2019). Amplicon sequence variants (ASVs) with >1% relative abundance in the bacterial community were taxonomically assigned using the EZBioCloud 16S-based identification service (Yoon *et al.*, 2017a).

### Bacterial isolation from asymptomatic sweet potato

To isolate bacteria, 50 g of asymptomatic sweet potato tubers harvested from field CH was added to 350 mL of plant-homogenizing solution (50 mM Tris-HCl, pH 7.0, 0.85% NaCl) supplemented with 35 μL of 2-mercaptoethanol and thoroughly homogenized using a blender. The homogenate was centrifuged at 8,000 rpm (SRX-201 centrifuge with a 9N rotor; TOMY SEIKO) at 10°C for 10 min. The resulting pellet was resuspended in plant-homogenizing solution, and the suspension was filtered through Miracloth (Merck KGaA). The filtrate was centrifuged at 500 rpm (CF16RX centrifuge with a T11A21 rotor; Hitachi) at 10°C for 5 min to remove residual plant debris. The supernatant containing bacterial cells was subsequently centrifuged at 10,000 rpm at 10°C for 5 min. The pellet was resuspended in 0.85% NaCl solution and mixed with an equal volume of glycerol stock solution (0.85% NaCl and 50% glycerol [v/v]). The suspension was stored at –80°C.

The glycerol stock solution was thawed and serially diluted ten-fold to 10^–6^ in 0.85% NaCl solution. Aliquots (100 μL) of each dilution were mixed with 40 μL of cycloheximide solution (25 mg mL^–1^ in 50% ethanol) and spread onto “Daigo” R2A agar plates (FUJIFILM Wako Pure Chemical Corporation). The plates were incubated at 30°C for 2 days. Single colonies were subsequently picked and purified, yielding a total of 40 bacterial isolates.

### Partial 16S rRNA gene sequencing of bacterial isolates

The isolates were grown in “Daigo” R2A broth at 30°C. After 24 h, 900 μL of the culture was centrifuged at 10,000 rpm (CF16RX centrifuge T11A21 rotor; Hitachi) at 10°C for 5 min. Genomic DNA was extracted from the bacterial pellet with an ISOPLANT DNA extraction kit (Nippon Gene) and an EconoSpin IIa mini spin column (Funakoshi). The 16S rRNA gene sequence was amplified by PCR with the primers 27F (5′-AGAGTTTGATCCTGGCTCAG-3′) (Lane, 1991) and 1492R (5′-GGTTACCTTGTTACGACTT-3′) (Turner *et al.*, 1999). The PCR mixture (50 μL) contained 0.25 μL of ExTaq HS DNA polymerase (TaKaRa Bio), 5 μL of 10× buffer, 4 μL of the dNTP mixture (2.5 mM each: TaKaRa Bio), 1 μL of each primer (50 μM), 2 μL of genomic DNA solution (10 ng μL^–1^), and sterilized water (up to the final volume). Cycling conditions were 98°C for 1 min and then 25 cycles of 98°C for 10 s, 60°C for 30 s, and 72°C for 1 min. PCR products were purified with AMPure XP beads according to the manufacturer’s protocol (Beckman Coulter). The PCR product was sequenced with the 27F primer or 1492R primer on a Big Dye Terminator v.‍ ‍3.1 sequencer and purified with a BigDye X Terminator Purification Kit according to the manufacturer’s protocol (Thermo Fisher Scientific). The products were analyzed on an ABI3500xl Genetic Analyzer. The partial sequence of the 16S rRNA gene of each isolate was taxonomically assigned against the EZBioCloud database.

### Local BLAST anal­ysis: identification of bacterial isolates that were abundant on asymptomatic sweet potato

ASVs with >1% relative abundance in the bacterial community were subjected to a BLAST search against the sequence library of the isolates, which was constructed in the present study using Genetyx Network Edition v. 16.0.3 software (GENETYX). Isolates with 100% sequence identity to the ASVs were further analyzed to complete the full length of the 16S rRNA gene; sequences read by the 27F, 520F (5′-GTGCCAGCAGCCGCGG-3′) (Namba *et al.*, 1993), and 520R (5′-ACCGCGGCTGCTGGC-3′) (Namba *et al.*, 1993) primers were assembled into one contig using ATGC ver.10 in Genetyx Network Edition software ver.16.0.3. The isolates were identified by using the almost full-length 16S rRNA gene sequence against the EZBioCloud database.

### Genome sequencing and genome-based identification

The genomic DNA of bacterial isolates was extracted with the ISOPLANT DNA extraction kit according to the manufacturer’s protocol. DNA solutions were sent to Macrogen Japan for PacBio Sequel II sequencing and *de novo* assembly of the reads. A Microbial Genome Assembly application (SMRTlink v13.0.0.207600) was used for assembly using HiFi reads. The mapping of reads against assembled contigs and polishing using Racon generated a consensus sequence of a higher quality. The complete genome sequence was analyzed using the DDBJ Fast Annotation and Submission Tool (DFAST; https://dfast.ddbj.nig.ac.jp/) (Tanizawa *et al.*, 2019). Average nucleotide identity (ANI) values were calculated using the ANI Calculator (Yoon *et al.*, 2017b) integrated within DFAST and compared with those of closely related strains. Digital DNA–DNA hybridization (dDDH) values were estimated using the Type (Strain) Genome Server (TYGS; https://tygs.dsmz.de/) (Freese *et al.*, 2026). A genome-based phylogenetic tree was constructed using TYGS. Since the genome sequence of‍ ‍*Enterobacter quasihormaechei* (GCA_004331385.1) was not included in the default TYGS dataset, this genome was additionally included in the phylogenetic anal­ysis.

### Isolation and tentative identification of fungi from diseased sweet potato

Fungi were isolated from diseased sweet potato on sweet potato agar (SPA) plates. To prepare SPA plates, asymptomatic sweet potato was weighed and homogenized with twice its weight in water in a blender. The mixture was filtered through a Miracloth. The filtrate was mixed with agar (final concentration of 1.5%) and then autoclaved. Streptomycin (cat. No. 194-08512; FUJIFILM Wako Pure Chemical Corporation) and Rose Bengal (cat. No. 30237-32; NACALAI TESQUE) were added to a final concentration each of 50 μg mL^–1^. The diseased sweet potato was cut into small pieces and placed onto the SPA plates. After 2 weeks of culture at 30°C, fungi formed giant colonies. The agar was cut into 5×5 mm squares at the edge of a colony and the fungal plug was transferred into the center of a fresh SPA plate. This passage-culture treatment was performed at least 3 times to obtain pure fungal cultures. Fungal isolates were maintained on potato dextrose agar (PDA) plates.

Fungal isolates were identified from the sequence of their internal transcribed spacer (ITS) region (White *et al.*, 1990). A 5×5 mm fungal plug was taken from a culture and inoculated into 20 mL of yeast glucose medium (YG medium) in an Erlenmeyer flask (cat. No. 4980FK100; AGC Techno Glass) that was then‍ ‍capped with a silicone plug (cat. No. 6-343-08; AS ONE Corporation). The YG medium comprised 5 g of yeast extract and 10 g of glucose in 1 L, and its pH was adjusted to 7.0 before autoclaving. The flask was incubated on a rotary shaker (cat. No. MMS-1020; TOKYO RIKAKIKAI) at 140 rpm at 30°C for 7 days. The culture was transferred into a 50-mL tube and centrifuged at 10,000 rpm (Himac CF16RX centrifugation machine and T11A21 rotor; Hitachi) for 5 min. The cell pellet was added to a Lysing Matrix E tube (MP Biomedicals), and genomic DNA was extracted as described in our previous study (Nakamura *et al.*, 2020). The concentration of the genomic DNA solution was measured on a NanoDrop-8000 spectrophotometer (Thermo Fisher Scientific). The ITS region was amplified by PCR with the primers ITS4 (5′-TCCTCCGCTTATTGATATGC-3′) and ITS5 (5′-GGAAGTAAAAGTCGTAACAAGG-3′) (White *et al.*, 1990). The almost full-length sequence of the ITS region was amplified and sequenced as described above for the 16S rRNA gene, except that the PCR annealing temperature was set to 53°C. Fungal isolates were identified through a BLASTN anal­ysis on the NCBI website (https://www.ncbi.nlm.nih.gov/) and by constructing a phylogenetic tree based on ITS region sequences. The tree incorporated diverse ITS sequences reported in studies on sweet potato diseases (Gai *et al.*, 2016; Lee *et al.*, 2019; Paul *et al.*, 2019) and was generated using MEGA v.11 software (Kumar *et al.*, 2018).

### Pathogenicity test of *D. destruens* on sweet potato tubers

One of the *D. destruens* isolates, designated 2YO, was cultured in 20 mL of YG medium as described above. Ten beads (cat. no. 314-06251, Bac’n’ Roll Beads; Nippon Gene) were added to the culture in a 50-mL tube, which was then mixed vigorously for 5 min using a mixer (cat. no. SE-04; TAITEC). The mixture was filtered through a 100-μm filter (BMA-CS100; BM Equipment), and the filtrate was centrifuged at 10,000 rpm at 10°C for 5 min. The resulting pellet was resuspended in 40 mL of sterilized water and used as the fungal inoculum. A total of 300 mL of vermiculite (Setogahara Kaen) was placed into a 500-mL autoclavable beaker (cat. no. 1044E; Sanplatec), covered with aluminum foil, and autoclaved. The inoculum was added to the vermiculite and mixed thoroughly using a sterilized spoon. The inoculated vermiculite was transferred into an M-sized Ziplock^®^ plastic bag (Asahi Kasei). Four ‘Narutokintoki’ sweet potato tubers (SSS size; Seibu-Seika) were cut and added to the bag, with the cut surfaces being completely buried in the vermiculite. The bags were incubated at 25°C for 2 weeks. In addition, *D. destruens* MAFF 247516, obtained from the NARO Genebank (Tsukuba, Japan), was tested.

### Dual culture assay of bacterial isolates for antagonistic activity against *D. destruens*

Bacterial isolates showing 100% sequence identity to the abundant ASV were cultured on R2A agar plates at 30°C overnight. *D. destruens* 2YO and MAFF 247516 were used and cultured on PDA plates (cat. no. 05709; Shimadzu Diagnostics) at 30°C for 4 weeks. A 5×5 mm fungal plug was placed at the center of a PDA plate, and the bacterial strains were streaked in a square surrounding the plug to perform a dual culture assay. The plates were incubated at 30°C for 2 weeks. A *D. destruens* plate without bacterial streaking was used as a control. Assays were performed in quadruplicate (*n*=4).

To investigate the secretion of fungal growth inhibitors by the bacteria, cell-free (CF) cultures (PDA-CF) were prepared. To prepare CF cultures, (1) bacterial cultures grown in R2A liquid medium at 30°C overnight were centrifuged at 10,000 rpm (CF16RX centrifuge, T11A21 rotor; Hitachi) at 10°C for 5 min, and the supernatants were filtered through a 0.2-μm cellulose acetate filter (cat. no. S6534-FMOSK, Minisart; Sartorius Japan); and (2) the filtrates were mixed with autoclaved PDA to a final concentration of 20% (v/v). A 5×5 mm fungal plug of *D. destruens* 2YO was placed at the center of each PDA-CF plate, and the plates were incubated at 30°C for 2 weeks. The assays were performed in quadruplicate (*n*=4).

## Results

### Tuber-associated microbial community of asymptomatic sweet potatoes grown in foot rot-infested fields

Thirteen bacterial taxa were detected at >1% relative abundance in asymptomatic sweet potato roots across the four fields (Table 1). Among these, ASV-129 (427 bp) was dominant in all fields, with an average relative abundance of 49.4%. The closest related species was *Enterobacter cancerogenus* (100% sequence identity to FYBA01000020). The second most abundant ASV overall was ASV-257 (422 bp), accounting for 7.4% of total relative abundance. This ASV showed 100% sequence identity to *Sphingobacterium multivorum* (AB100738) and was dominant only in field AH, where it reached a relative abundance of 28.4%. The third most abundant ASV overall was ASV-186 (427 bp), representing 6.4% of total relative abundance. The closest related species was *Pseudomonas plecoglossicida* (100% sequence identity to BBIV01000080). The only other ASV detected at a relative abundance >1% in asymptomatic roots across all four fields was ASV-87 (427 bp), which showed 100% sequence identity to *Delftia tsuruhatensis* (BCTO01000107).

### Tentative identification of bacterial isolates abundantly present in asymptomatic sweet potato tubers

Forty bacterial strains were isolated from asymptomatic sweet potatoes grown in field CH (Table 2). Among these, nine isolates (7CK, 12CK, 19CK, 20CK, 28CK, 34CK, 36CK, 37CK, and 38CK) showed 100% sequence identity to the dominant ASV-129. A sequence anal­ysis of the almost full-length 16S rRNA genes showed that these strains had‍ ‍100% sequence identity with both *E. hormaechei* subsp.‍ ‍*xiangfangensis* and *E. quasihormaechei*. Since the 16S rRNA gene sequence of *E. hormaechei* subsp. *xiangfangensis* is identical to that of *E. quasihormaechei*, it was not possible to distinguish between the two species for these nine isolates. In addition, four isolates (9CK, 18CK, 31CK, and 40CK) showed 100% sequence identity to ASV-39, which was the 11th most abundant ASV detected in asymptomatic sweet potato roots (Table 1). These four isolates were identified as *Stenotrophomonas maltophilia*.

### Genome characterization of *Enterobacter* sp. 12CK

**Fig. 1.**
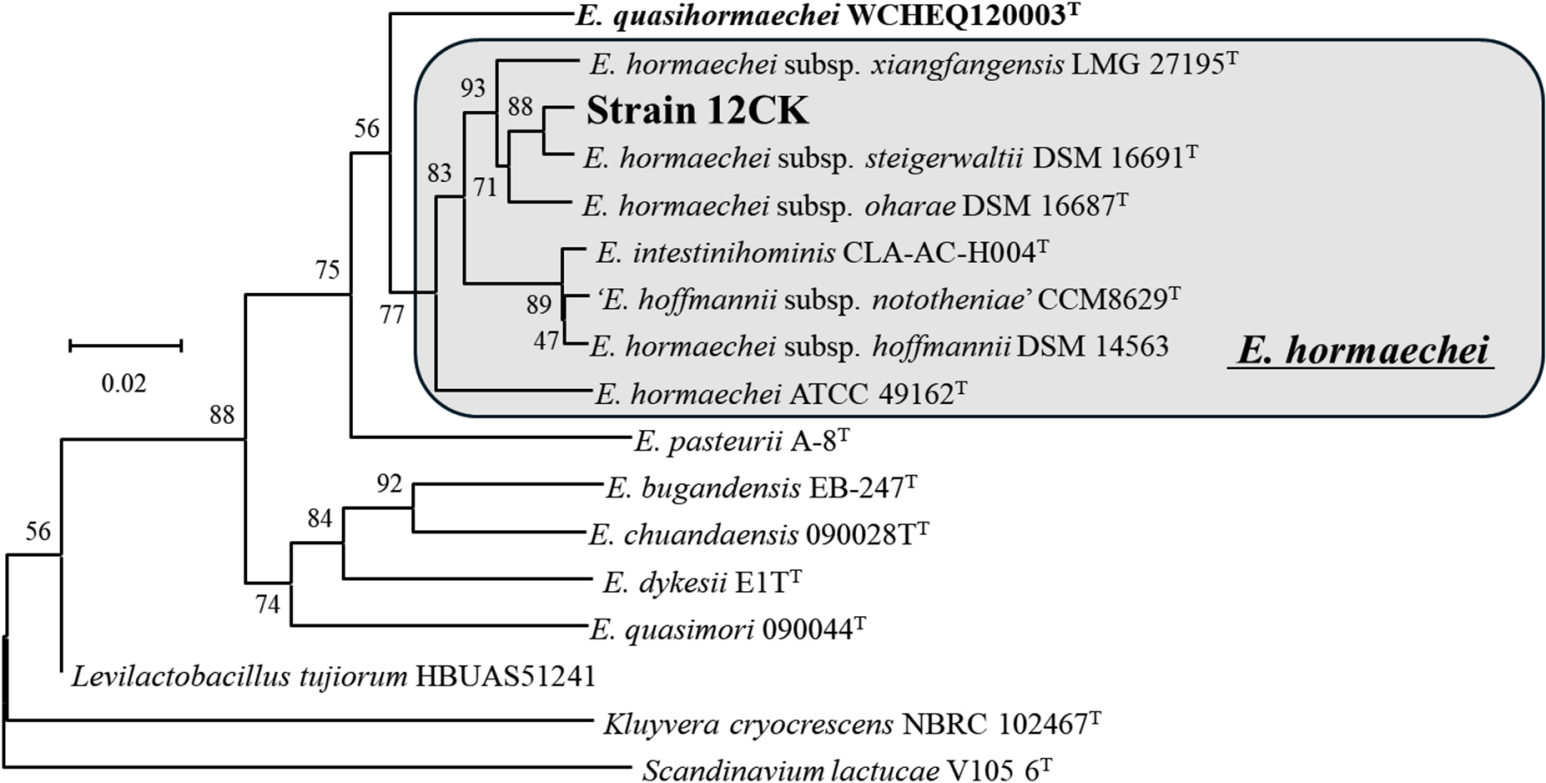
Genome-based phylogenetic tree of *Enterobacter* strains. Tree inferred with FastME 2.1.6.1 (Lefort *et al.*, 2015) from GBDP distances calculated from genome sequences. Branch lengths are scaled in terms of GBDP distance formula d5. The numbers above branches are GBDP pseudo-bootstrap support values >60% from 100 replications, with an average branch support of 81.4%. The tree was rooted at the midpoint (Farris, 1972). The description of the tree is quoted from the TYGS anal­ysis output. Since the genome sequence of *Enterobacter quasihormaechei* (GCA_004331385.1) was not included in the default TYGS dataset, it was additionally incorporated into the phylogenetic anal­ysis.

The complete genome of strain 12CK comprised 4,832,483 bp. The ANI anal­ysis identified strain 12CK as *E. hormaechei* (Table 3). All type strains fulfilling the species thresholds of ANI >95% and dDDH (d4) >70% (Riesco and Trujillo, 2024) belonged to *E. hormaechei*, whereas strain 12CK showed lower values—ANI 93.64% and dDDH 53.0%—with *E. quasihormaechei* WCHEQ120003^T^, indicating that it is not *E. quasihormaechei* and needs to be identified as a distinct species. The genome-based phylogenetic anal­ysis placed strain 12CK within the *E. hormaechei* clade, which also included *E. intestinihominis* CLA-AC-H004^T^ as an exception, and was clearly distinct from *E. quasihormaechei* (Fig. 1), further supporting its identification as *E. hormaechei*. The DFAST anal­ysis revealed that strain 12CK harbored eight copies of the 16S rRNA gene, all showing 100% sequence identity to the dominant ASV-129.

### Isolation of *D. destruens*

**Fig. 2.**
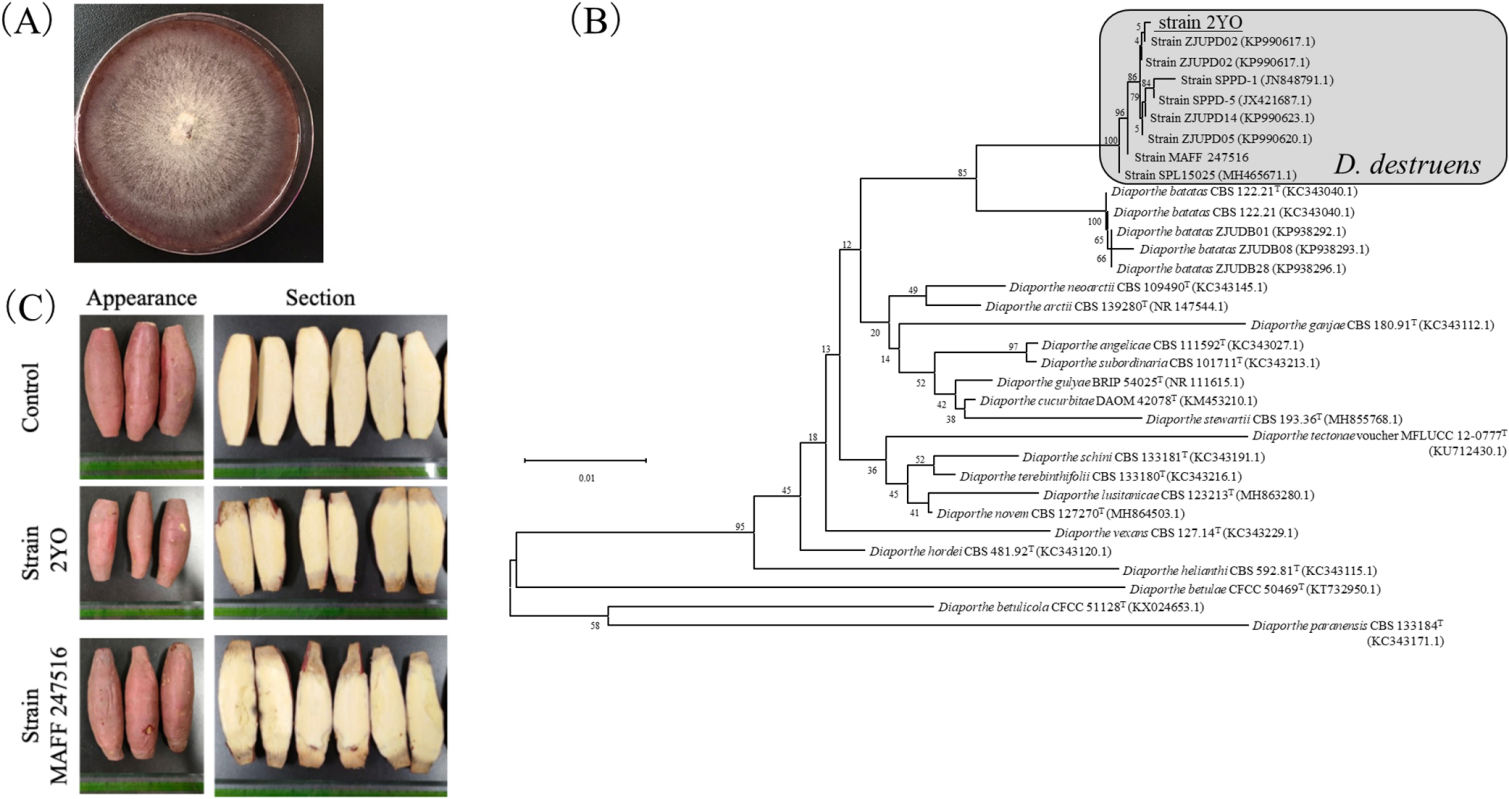
Isolation of *Diaporthe destruens* 2YO from disease sweet potato. (A) Colony morphology of strain 2YO grown on sweet potato agar (SPA) plates at 30°C for 2 weeks. (B) Phylogenetic anal­ysis based on ITS sequences. A gray-shaded box indicates the cluster formed by strains identified as *D. destruens*. Accession numbers are shown in parentheses. (C) Pathogenicity test showing symptoms on sweet potato tubers two weeks after the inoculation with strain 2YO; negative and positive control tubers were treated with sterile water and *D. destruens* MAFF 247516, respectively.

Strain 2YO was isolated from a diseased sweet potato. The colony morphology of this strain on SPA plates is shown in Fig. 2A. The nearly full-length ITS sequence of‍ ‍strain 2YO showed 100% sequence identity to that of‍ ‍*D.‍ ‍destruens* SPL18008 (GenBank accession no. MH465673.1). The phylogenetic anal­ysis based on ITS sequences showed that strain 2YO clustered with *D. destruens* strains associated with foot rot disease (Gai *et al.*, 2016; Lee *et al.*, 2019; Paul *et al.*, 2019) (Fig. 2B). Pathogenicity assays further confirmed that *D. destruens* strain 2YO caused foot rot disease in sweet potato (Fig. 2C).

### Ability of *E. hormaechei* 12CK to inhibit the growth of *D. destruens*

**Fig. 3.**
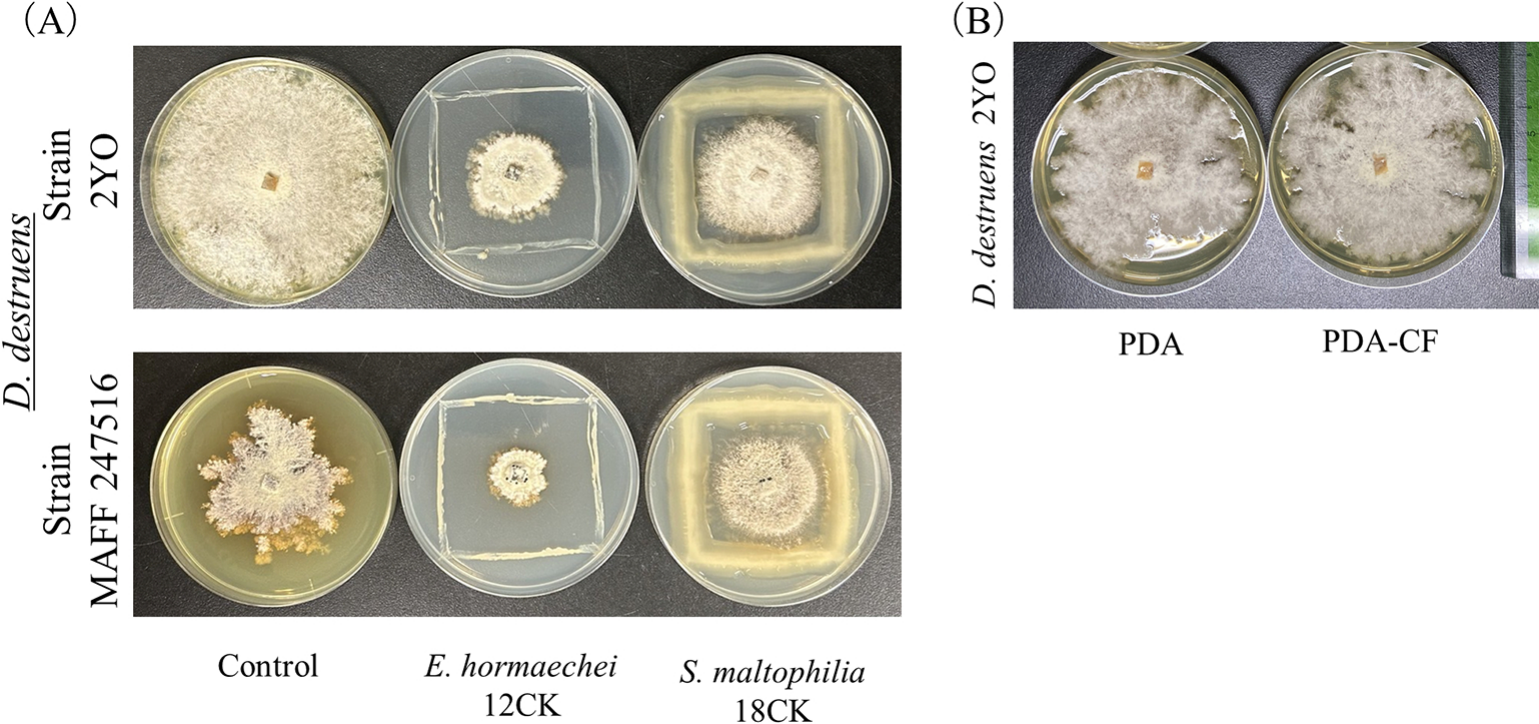
Inhibition of Diaporthe destruens growth by Enterobacter hormaechei 12CK. (A) Growth inhibition assay (dual culture assay) showing the antagonistic effects of strain 12CK against *D. destruens* 2YO on PDA medium. (B) Growth of *D. destruens* 2YO on PDA-CF medium (PDA containing the cell-free culture filtrate of strain 12CK).

*E. hormaechei* 12CK strongly inhibited, whereas *S. maltophilia* 18CK moderately suppressed the growth of *D. destruens* strains 2YO and MAFF 247516 on PDA medium (Fig. 3A). In a growth assay of *D. destruens* 2YO on PDA-CF medium, *E. hormaechei* 12CK did not inhibit the growth of *D. destruens* under the experimental condition used in the present study (Fig. 3B).

## Discussion

The most dominant bacterium in asymptomatic sweet potatoes (ASV-129) was identified as *Enterobacter* sp., with an average relative abundance of 49.4% (Table 1). The 16S rRNA gene sequence of *E. hormaechei* 12CK showed 100% identity to the dominant ASV-129; however, this strain possesses eight copies of the 16S rRNA gene. Therefore, the actual relative abundance of *Enterobacter* sp. may be approximately 10.9% when adjusted for the gene copy number. Nevertheless, *Enterobacter* sp. remains the most abundant bacterial taxon in asymptomatic sweet potatoes. Puri *et al.* (2019) reported that *Pseudomonas* was the dominant genus (36.7%), whereas *Enterobacteriaceae* represented the second most abundant bacterial group (11.9%) in “healthy” sweet potatoes grown under both pot and open-field conditions. Collectively, these findings suggest that *Enterobacter* spp. are consistently abundant in sweet potato tissues. The markedly increased relative abundance of *Enterobacter* spp. observed in asymptomatic tubers from foot rot-infested fields may reflect pathogen pressure. As described in the Introduction, dominant bacteria in asymptomatic tubers adjacent to infected plants may serve as candidates for disease control (Cao *et al.*, 2020). Consistent with this concept, we demonstrated that *E. hormaechei* 12CK strongly inhibited the growth of *D. destruens*. Unlike *Bacillus* strains reported by Mateus *et al.* (2021), the culture supernatant of strain 12CK did not inhibit the growth of *D. destruens* under the conditions tested in the present study. Therefore, the observed suppression may be attributable to competition for nutrients. The type strain of *E. hormaechei* subsp. *xiangfangensis*, which is the closest relative of strain 12CK based on 16S rRNA gene and whole-genome sequence anal­yses, was originally isolated from an infected surgical wound of a patient with tonsillar carcinoma (Hoffmann *et al.*, 2005). To date, members of *E. hormaechei* have been isolated from a wide range of environments, including soil, water, plants, insects, and animal gastrointestinal tracts (Yeh *et al.*, 2022). This species has been reported to exhibit antagonistic activity, either *in vitro* or *in vivo*, against *Fusarium oxysporum* f. sp. *radicis lycopersici*, the causal agent of tomato *Fusarium* wilt (Bendaha and Belaouni, 2019); *Colletotrichum falcatum*, which causes red rot disease of sugarcane (Hajialigol *et al.*, 2023); and *Phytophthora capsici*, the causal agent of Phytophthora blight of *Capsicum* spp. (Admassie *et al.*, 2023). In addition, *E. hormaechei* is known to promote plant growth (Inui *et al.*, 2012; Roslan *et al.*, 2020; Ranawat *et al.*, 2021a, 2021b). These characteristics suggest the benefits of applying *E. hormaechei* 12CK as a microbial inoculant. Evaluations of its ability to suppress foot rot disease under pot and field conditions will be an important next step. *Pseudomonas*, *Bacillus*, and *Burkholderia* species have been extensively studied as agricultural biocontrol agents or plant growth-promoting bacteria; however, some members of these genera are recognized as opportunistic pathogens. Similarly, *E. hormaechei* has been reported as a nosocomial and opportunistic human pathogen (Yeh *et al.*, 2022; Hajialigol *et al.*, 2023). In addition, *S. maltophilia*, which exhibited moderate inhibitory activity in the present study, is also widely recognized as an opportunistic human pathogen (Kumar *et al.*, 2023). Therefore, an assessment of potential human pathogenicity and biosafety will be necessary prior to the practical application of *E. hormaechei* 12CK and *S. maltophilia* 18CK.

To develop effective disease control strategies, it is essential to establish a rapid and reliable method for pathogenicity testing (Nomiyama *et al.*, 2022). Conventionally, fungal inocula (conidial suspensions) are prepared using labor-intensive procedures (Gai *et al.*, 2016; Fujiwara *et al.*, 2021; Maeda *et al.*, 2022), which typically involve culturing *D. destruens* on PDA plates, inducing sporulation by black-light irradiation, harvesting spores by scraping with sterile water, and filtering the suspension through gauze. In the present study, as described in the Materials and Methods, we developed a simplified pathogenicity assay based on liquid culture. Briefly, *D. destruens* was cultured in yeast extract–glucose (YG) liquid medium, and a fungal inoculum suitable for pathogenicity testing was prepared by bead agitation followed by filtration through a 100-μm mesh. The application of this inoculum, mixed with vermiculite, to wounded sweet potato tubers reproducibly induced disease symptoms within two weeks. This simplified method provides a practical alternative for pathogenicity assays and may facilitate future studies aimed at developing microbial control agents against *D. destruens*.

## Acknowledgements

The authors would like to thank ELSS (https://www.elss.co.jp/ja/) for the English language review. In addition, generative AI tools (ChatGPT and Microsoft Copilot) were used to assist in improving the clarity, grammar, and readability of the manuscript.

### Funding

This research was supported by a research grant for advanced studies from the United Graduate School of Agricultural Sciences, Kagoshima University; the JT SDGs Contribution Project; and the Akio Kiyokawa Scholarship Foundation.

### Competing interests

The authors declare no competing interests regarding the publication of this manuscript.

### Data availability

Raw reads used for the microbial community anal­ysis in this study have been deposited in the NCBI SRA database under accession numbers SRR31226875–SRR31226878. The 16S rRNA gene sequences of the bacterial isolates have been deposited in NCBI GenBank under accession numbers PX352430–PX352469. The whole-genome sequence of *E. hormaechei* 12CK has been deposited in the NCBI Genome database (BioProject ID: PRJNA1328955). The ITS sequence of *D. destruens* 2YO has been deposited in NCBI GenBank under the accession number PX393787. All other data that support the results of this study are available from the corresponding author upon reasonable request.

## Citation

Kubota, C., Hashimoto, M., Ougitani, Y., Yujima, R., Ishibashi, M., and Tsurumaru, H. (2026) Dominance of *Enterobacter* spp. in Asymptomatic Sweet Potato Tubers Grown in Foot Rot–infested Fields. *Microbes Environ ***41**: ME26007.

https://doi.org/10.1264/jsme2.ME26007

## Figures and Tables

**Table 1.** Composition of the microbial community associated with asymptomatic sweet potatoes

ASV			Blast result				Relative abundance (%)				
ASV No.	bp		Hit taxon name	Accession No.	Similarity (%)		AH	BH	CH	DH	Average
129	427		*Enterobacter cancerogenus*	FYBA01000020	100		30.5	26.4	63.8	76.8	49.4
257	422		*Sphingobacterium multivorum*	AB100738	100		28.4	0.0	0.0	1.09	7.4
186	427		*Pseudomonas plecoglossicida*	BBIV01000080	100		7.9	10.9	5.8	0.96	6.4
87	427		*Delftia tsuruhatensis*	BCTO01000107	100		1.4	19.0	1.04	3.5	6.3
20	422		*Chryseobacterium defluvii*	jgi.1085851	99.05		0.0	15.4	0.0	0.2	3.9
134	427		*Flavobacterium acidificum*	JX986959	100		0.0	2.4	3.9	0.3	1.6
99	407		*Curtobacterium luteum*	X77437	100		0.3	0.0	5.9	0.0	1.5
195	422		*Sphingobacterium multivorum*	AB100738	99.76		4.7	0.0	1.2	0.0	1.5
63	422		*Chryseobacterium arachidis*	jgi.1107773	100		5.7	0.0	0.0	0.0	1.4
255	428		*Bacillus tequilensis*	AYTO01000043	100		0.4	0.0	4.9	0.0	1.3
39	427		*Pseudomonas hibiscicola*	AB021405	99.77		0.3	1.5	1.15	1.4	1.1
196	427		*Pseudomonas putida*	AP013070	100		2.3	0.4	0.0	1.6	1.1
150	407		*Streptomyces jiujiangensis*	KF938657	100		0.3	3.1	0.0	0.8	1.1

**Table 2.** Identification of bacterial isolates from asymptomatic sweet potatoes based on a 16S rRNA gene anal­ysis ^a^

Strains	Blast results					Identical ASVs	
	Sequence length (bp)	Similarity (%)	Accession No.	Related species		ASV No.	identity
1CK	451	100	OQ852713	*Stenotrophomonas riyadhensis*			
2CK	817	99.9	BBIQ01000036	*Pseudomonas fulva*			
3CK	708	99.9	jgi.1102370	*Agrobacterium pusense*			
4CK	838	99.9	jgi.1102370	*Agrobacterium pusense*			
5CK	872	99.9	jgi.1102370	*Agrobacterium pusense*			
6CK	858	100	BBIQ01000036	*Pseudomonas fulva*			
7CK	1,460	99.8	FYBF01000083	*Enterobacter hormaechei* subsp*. xiangfangensis*/*E. quasihormaechei*		129	427/427 (100%)
8CK	838	99.9	JALV01000036	*Stenotrophomonas maltophilia/Stenotrophomonas pavanii*			
9CK	1,464	99.9	JALV01000036	*Stenotrophomonas maltophilia*		39	427/427 (100%)
10CK	765	99.9	jgi.1102370	*Agrobacterium pusense*			
11CK	918	100	BBIQ01000036	*Pseudomonas fulva*			
12CK	1,266	100	FYBF01000083	*Enterobacter hormaechei* subsp*. xiangfangensis*/*E. quasihormaechei*		129	427/427 (100%)
13CK	919	100	BBIQ01000036	*Pseudomonas fulva*			
14CK	853	100	BBIQ01000036	*Pseudomonas fulva*			
15CK	949	99.5	QKVM01000121	*Pseudomonas sichuanensis*			
16CK	866	99.2	EU912483	*Leifsonia lichenia*			
17CK	907	100	JALV01000036	*Stenotrophomonas maltophilia*			
18CK	1,284	99.8	JALV01000036	*Stenotrophomonas maltophilia*		39	427/427 (100%)
19CK	1,266	100	FYBF01000083	*Enterobacter hormaechei* subsp*. xiangfangensis*/*E. quasihormaechei*		129	427/427 (100%)
20CK	1,266	100	FYBF01000083	*Enterobacter hormaechei* subsp*. xiangfangensis*/*E. quasihormaechei*		129	427/427 (100%)
21CK	679	99.9	AP013070	*Pseudomonas putida*			
22CK	839	100	BBIQ01000036	*Pseudomonas fulva*			
23CK	846	100	BBIQ01000036	*Pseudomonas fulva*			
24CK	842	100	jgi.1102370	*Agrobacterium pusense*			
25CK	841	100	jgi.1102370	*Agrobacterium pusense*			
26CK	821	100	BBIQ01000036	*Pseudomonas fulva*			
27CK	841	100	jgi.1102370	*Agrobacterium pusense*			
28CK	1,458	99.9	FYBF01000083	*Enterobacter hormaechei* subsp*. xiangfangensis*/*E. quasihormaechei*		129	427/427 (100%)
29CK	958	99.9	JALV01000036	*Stenotrophomonas maltophilia/Stenotrophomonas pavanii*			
30CK	854	100	BBIQ01000036	*Pseudomonas fulva*			
31CK	1,473	99.9	JALV01000036	*Stenotrophomonas maltophilia*		39	427/427 (100%)
32CK	837	99.5	JALV01000036	*Stenotrophomonas maltophilia*			
33CK	784	99.4	BCUT01000013	*Variovorax paradoxus*			
34CK	1,393	99.9	FYBF01000083	*Enterobacter hormaechei* subsp*. xiangfangensis*/*E. quasihormaechei*		129	427/427 (100%)
35CK	914	99.9	JALV01000036	*Stenotrophomonas maltophilia/Stenotrophomonas pavanii*			
36CK	1,404	99.9	FYBF01000083	*Enterobacter hormaechei* subsp*. xiangfangensis*/*E. quasihormaechei*		129	427/427 (100%)
37CK	1,349	99.9	FYBF01000083	*Enterobacter hormaechei* subsp*. xiangfangensis*/*E. quasihormaechei*		129	427/427 (100%)
38CK	1,403	99.9	FYBF01000083	*Enterobacter hormaechei* subsp*. xiangfangensis*/*E. quasihormaechei*		129	427/427 (100%)
39CK	710	100	BBIQ01000036	*Pseudomonas fulva/Pseudomonas parafulva*			
40CK	1,487	99.3	JALV01000036	*Stenotrophomonas maltophilia*		39	427/427 (100%)

^a^ Gray indicates strains with 100% sequence identity to the abundant ASV.

**Table 3.** ANI and dDDH values of strain 12CK relative to closely related strains^a^

Organism name	Strain name	Relation to type	Accession No.	ANI (%)	dDDH (d4, in%)
*Enterobacter hormaechei* subsp. *steigerwaltii*	DSM 16691	type	GCA_001729725.1	98.81	91.0
*Enterobacter hormaechei* subsp. *oharae*	FDAARGOS 1533	type	GCA_020097195.1	97.69	80.3
*Enterobacter hormaechei* subsp. *xiangfangensis*	LMG 27195	type	GCA_001729785.1	97.28	76.1
*Enterobacter hormaechei* subsp. *hoffmannii*	DSM 14563	suspected type	GCA_001729745.1	96.05	66.8
*Enterobacter hormaechei* subsp. *hormaechei*	ATCC 49162	type	GCA_000213995.1	94.87	61.1
*Enterobacter quasihormaechei*	WCHEQ120003	type	GCA_004331385.1	93.64	53.0
*Enterobacter pasteurii*	A-8	type	GCA_028890245.1	91.57	43.5

^a^ Gray indicates strains with ANI >95% and dDDH (d4) >70%, the accepted thresholds for species delimitation (Riesco and Trujillo, 2024).
